# Escherichia coli B-Strains Are Intrinsically Resistant to Colistin and Not Suitable for Characterization and Identification of *mcr* Genes

**DOI:** 10.1128/spectrum.00894-23

**Published:** 2023-05-18

**Authors:** Anna Schumann, Alexa R. Cohn, Ahmed Gaballa, Martin Wiedmann

**Affiliations:** a Department of Food Science, Cornell University, Ithaca, New York, USA; b Graduate Field of Biomedical and Biological Sciences, Cornell University, Ithaca, New York, USA; The University of Sydney

**Keywords:** *E. coli* B-strains, colistin, *mcr* genes

## Abstract

Antimicrobial resistance is an increasing threat to human and animal health. Due to the rise of multi-, extensive, and pandrug resistance, last resort antibiotics, such as colistin, are extremely important in human medicine. While the distribution of colistin resistance genes can be tracked through sequencing methods, phenotypic characterization of putative antimicrobial resistance (AMR) genes is still important to confirm the phenotype conferred by different genes. While heterologous expression of AMR genes (e.g., in Escherichia coli) is a common approach, so far, no standard methods for heterologous expression and characterization of *mcr* genes exist. E. coli B-strains, designed for optimum protein expression, are frequently utilized. Here, we report that four E. coli B-strains are intrinsically resistant to colistin (MIC 8–16 μg/mL). The three tested B-strains that encode T7 RNA polymerase show growth defects when transformed with empty or *mcr*-expressing pET17b plasmids and grown in the presence of IPTG; K-12 or B-strains without T7 RNA polymerase do not show these growth defects. E. coli SHuffle T7 express carrying empty pET17b also skips wells in colistin MIC assays in the presence of IPTG. These phenotypes could explain why B-strains were erroneously reported as colistin susceptible. Analysis of existing genome data identified one nonsynonymous change in each *pmrA* and *pmrB* in all four E. coli B-strains; the E121K change in PmrB has previously been linked to intrinsic colistin resistance. We conclude that E. coli B-strains are not appropriate heterologous expression hosts for identification and characterization of *mcr* genes.

**IMPORTANCE** Given the rise in multidrug, extensive drug, and pandrug resistance in bacteria and the increasing use of colistin to treat human infections, occurrence of *mcr* genes threatens human health, and characterization of these resistance genes becomes more important. We show that three commonly used heterologous expression strains are intrinsically resistant to colistin. This is important because these strains have previously been used to characterize and identify new mobile colistin resistance (*mcr*) genes. We also show that expression plasmids (i.e., pET17b) without inserts cause cell viability defects when carried by B-strains with T7 RNA polymerase and grown in the presence of IPTG. Our findings are important as they will facilitate improved selection of heterologous strains and plasmid combinations for characterizing AMR genes, which will be particularly important with a shift to Culture-independent diagnostic tests where bacterial isolates become increasingly less available for characterization.

## INTRODUCTION

Antimicrobial resistance (AMR) is an increasing problem and has been declared one of the top 10 global public health threats to humanity by the World Health Organization (WHO) (https://www.who.int/news-room/fact-sheets/detail/antimicrobial-resistance). As AMR spreads and more bacteria become multidrug, extensively drug, or pandrug resistant, our current antibiotics become ineffective and new drugs are needed to combat infections (1, https://www.who.int/news-room/fact-sheets/detail/antimicrobial-resistance). Unfortunately, new drugs are lacking, so health care providers increasingly rely on last-resort antibiotics like colistin (2–5). Colistin, also called polymyxin E, is a cationic antimicrobial peptide discovered in 1947 (5, 6). Even though colistin is nephro- and neurotoxic, colistin use in human medicine has increased in recent years and colistin has been defined as a “critically important antimicrobial for human medicine” by WHO (3, 4, 7, 8).

Colistin’s activity is narrow and it is effective against some members of the order *Enterobacterales* and the species Pseudomonas aeruginosa, while other *Enterobacterales* like Proteus spp. are resistant (4, 5). Colistin has a positively charged head-group, which is electrostatically attracted to the negatively charged phosphate groups of lipopolysaccharide (LPS) molecules within the outer membrane (OM) of Gram-negative bacteria (3, 4). Colistin competitively displaces the Mg^2+^ and Ca^2+^ ions that normally surround the LPS molecules to stabilize the OM (3, 4). This displacement disrupts the OM and causes the leakage of cellular material, lysis, and cell death (3, 5, 9).

Colistin resistance was discovered soon after initiation of its use as an antibiotic. Originally it was thought that resistance was only conferred by chromosomal mutations in genes regulating expression of LPS modifying enzymes (6, 10). More specifically, modifications made by enzymes EptA and ArnT, which transfer phosphoethanolamine or 4-amino-4-deoxy-l-arabinose (l-Ara4N), respectively, to the lipid A portion of LPS, can reduce the negative charge of lipid A, thus blocking colistin’s electrostatic interaction with LPS (6, 10–12). The production of EptA and ArnT is regulated by two separate two-component systems PhoPQ and PmrAB; missense mutations in *phoPQ* and *pmrAB* can cause constitutive activation of lipid-modifying enzymes that result in lipid A modification and thus colistin resistance (6, 11, 13–16). However, in 2015 the first plasmid-borne gene providing colistin resistance, named mobile colistin resistance (*mcr*) gene, was discovered (17); this gene encodes a protein with a structure similar to EptA. Since then nine more variants (i.e., *mcr-2* to *mcr-10*) and many more subvariants (e.g., *mcr-2.2*) have been discovered (18–26).

To determine whether a given gene confers colistin resistance and to assess the level of colistin resistance conferred, authors oftentimes express *mcr* genes in heterologous laboratory Escherichia coli strains using either the native plasmid (17, 27, 28) or cloning the gene into a plasmid downstream of an inducible promoter like the T7 RNA polymerase promoter with the *lac* operator (e.g., pET plasmids) (29, 30). This method is necessary when discovering new variants from whole-genome sequence (WGS) data and when the isolate is not available or culturable. Variants identified in genome databases can be synthesized and characterized through expression in heterologous expression hosts. However, many different strain and plasmid combinations have been used in the past, making it difficult to compare resistance levels obtained from different heterologous expression systems (7, 31). E. coli B-strains and their derivatives are frequently used as hosts to express recombinant proteins because they were engineered to optimize protein production through their lack of flagella, encoding few proteases, and having increased amino acid biosynthesis (32, 33). Genetically B- and K-12 strains are very similar; for example, B-strain REL60 and K-12 strain MG1655 share over 99.1% nucleotide identity (33). The term E. coli B was first used by Delbrück and Luria in 1942 (34). E. coli BL21 is a descendant of that strain, created through strain exchanges between laboratories, mutagenesis and P1 transductions (34). Detailed information of the derivation of the strain can be found elsewhere (34).

As part of efforts to create a standardized expression system for identification and characterization of *mcr* genes, we noticed that several E. coli B-strains appeared to be intrinsically resistant to colistin, despite that E. coli B-strains (e.g., BL21[DE3]) have frequently been used to characterize *mcr* genes (29, 30). We thus performed a comprehensive analysis of multiple parent E. coli B-strains as well as corresponding strains with the pET17b expression plasmid with and without a cloned *mcr-3*-*FLAG*, which show that E. coli B-strains are intrinsically resistant to colistin possibly due to mutations in *pmrAB*. We also identified substantial toxicity to the E. coli host strains that contain the pET17b plasmid in the presence of IPTG. Combined, our data strongly suggest that E. coli B-strains, particularly when used with pET plasmids, may not be appropriate to identify and characterize *mcr* genes.

## RESULTS

### Four E. coli B-strains show intrinsic resistance to colistin, while two K-12 strains show sensitivity to colistin.

During preliminary experiments assessing different host-strain-plasmid combinations for their suitability for heterologous expression of *mcr* genes, the tested B-strains (i.e., BL21[DE3], T7 express lysY/l^q^) showed high levels of resistance to colistin (8 to 16 μg/mL). For reference, the Clinical and Laboratory Standards Institute (CLSI) defines colistin resistance of *Enterobacterales* as an MIC of ≥4 μg/mL (35). Based on these findings as well as some previous reports (36, 37), we hypothesized that B-strains are intrinsically resistant to colistin, while K-12 strains are not. To test this hypothesis, we performed a MIC assay on four E. coli B-strains, (i) BL21, (ii) BL21(DE3), (iii) T7 express lysY/l^q^ (abbreviated as “T7”), and (iv) SHuffle T7 express (abbreviated as “SHuffle”) as well as two E. coli K-12 strains, (i) NEB5α and (ii) Top10; all data were collected in parent strains without expression plasmid grown in media without IPTG. While the four B-strains showed MICs of 8 μg/mL (BL21 and BL21[DE3]) and 16 μg/mL (T7 and SHuffle), both K-12 strains showed MICs of <0.5 μg/mL (Table 1).

**TABLE 1:** MICs of B- and K-12 strains

Strain	Lineage	MIC (μg/mL)^*a*^
E. coli BL21	B	8
E. coli BL21(DE3)	B	8
E. coli T7 express lysY/l^q^	B	16
E. coli SHuffle T7 express	B	16
E. coli NEB5α	K-12	<0.5
E. coli Top10	K-12	<0.5

a MIC values are based on three biological replicates (except for SHuffle where *n* = 2); in all cases all replicates yield the same MIC value.

### Relative to E. coli K-12, E. coli B-strains show nonsynonymous substitutions in *pmrA* and *pmrB*, two genes known to contribute to colistin resistance.

**FIG 1:**
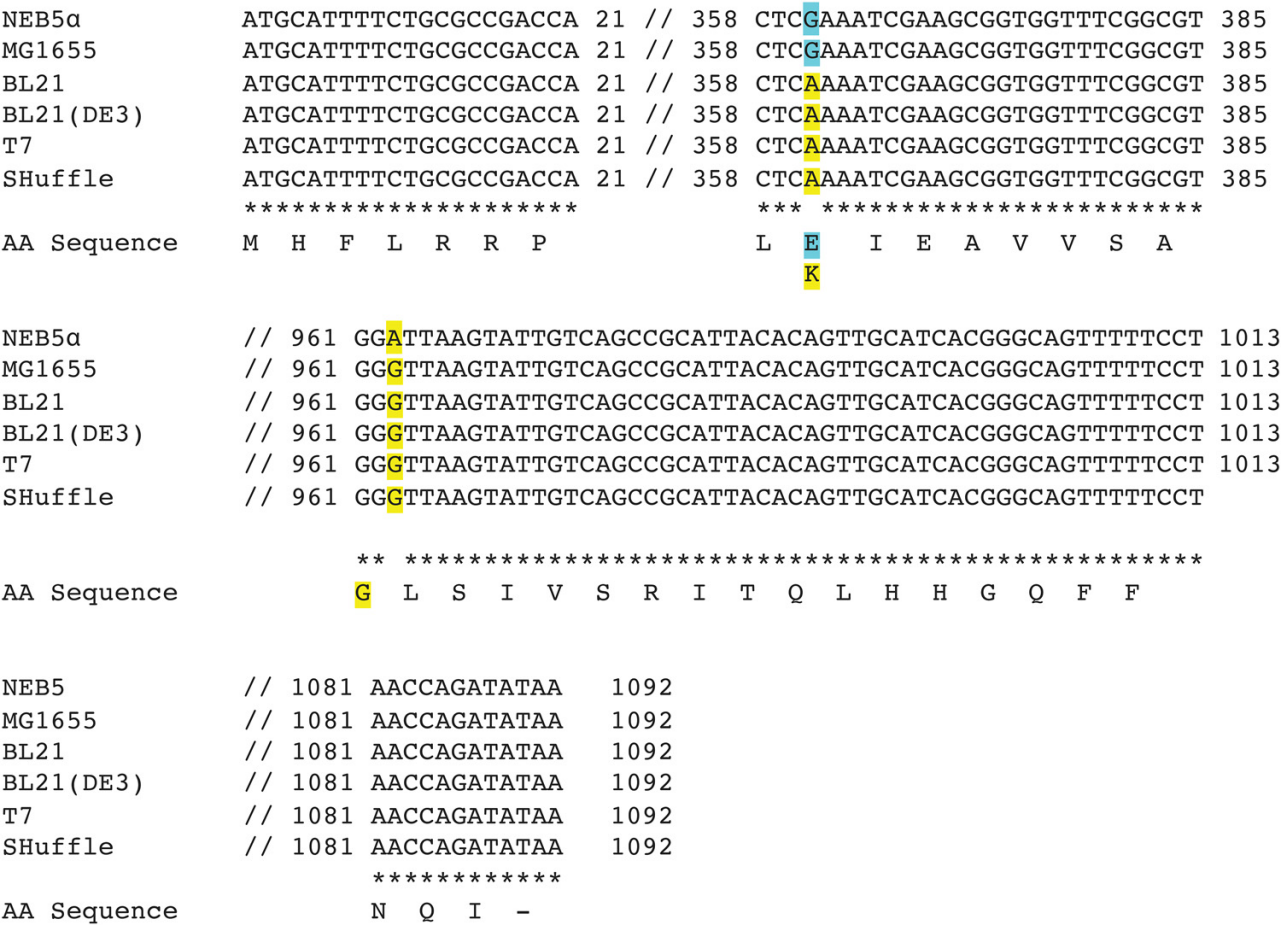
Alignment of *pmrB* DNA sequences of E. coli B- and K-12 strains used in this study. Alignment of genes and matching amino acid sequences was performed by Clustal Omega (66). Sequences from nucleotides 22–357, 386–960, and 1014–1080 were omitted because the strains shared 100% sequence identity for each of these regions. Base changes are highlighted; coloring indicates which base change correlates with which amino acid. Refer to Materials and Methods section for details on how the sequences were acquired. T7, T7 express lysY/l^q^; SHuffle, SHuffle T7 express.

Previous papers have reported mutation hot spots in *pmrA* and *pmrB* that have been linked to colistin resistance (15, 36). We thus hypothesized that the increased colistin MIC values observed here in the four E. coli B-strains, compared to the two K-12 strains, could be due to mutations in *pmrA* and/or *pmrB*. To test this hypothesis, we aligned *pmrA* and *pmrB* of the four B-strains and K-12 strain NEB5α to *pmrA* and *pmrB* of E. coli K-12 MG1655 (as a reference strain); E. coli Top10 *pmrA* and *pmrB* are not included in this alignment because Top10’s genome sequence is not publicly available. The *pmrA* alignment identified a total of seven synonymous and one nonsynonymous site over the 669 nucleotides (nt) length of the coding sequence (Fig. S1). For all eight sites, all four E. coli B-strains showed the same sequences and both NEB5α and MG1655 showed the same sequences, consistent with their classification into different E. coli lineages (i.e., B and K-12). For the nonsynonymous substitution at amino acid site 29, the four B-strains encoded a glycine, while the two K-12 strains encoded a serine (Fig. S1). The *pmrB* alignment identified one synonymous and one nonsynonymous site over the 1,092 nt length of the coding sequence (Fig. 1); for the nonsynonymous site, all four E. coli B-strains showed the same sequence, encoding a lysine, while both K-12 strains encoded a glutamate (Fig. 1). For the synonymous substitution, NEB5α was the only strain that deviated from the consensus DNA sequence (Fig. 1).

### IPTG-induced E. coli B-strains carrying pET17b-*mcr-3-FLAG* show 2-fold higher colistin MIC values compared to empty plasmid controls.

As B-strains have been used in a few studies to characterize *mcr* genes, despite evidence for intrinsic colistin resistance, we generated three different lineage B-strains (i.e., BL21[DE3], T7, SHuffle) that each either carry a pET17b plasmid expressing *mcr-3-FLAG* (“pET17b-*mcr-3*”) or an empty pET17b plasmid. We chose to use *mcr-3* because the colistin MIC values of heterologous strains expressing *mcr-3* are well characterized and should be 2 to 4 μg/mL, depending on the heterologous expression system used and *mcr-3* subvariant (24, 38, 39). Under induction (either 0.4 or 1 mM IPTG), BL21(DE3) and T7 strains expressing *mcr-3* showed 2-fold higher colistin MIC values compared to the corresponding strains carrying an empty plasmid (Table 2). For example, BL21(DE3) expressing *mcr-3* showed MIC values of 8 μg/mL (for both 0.4 and 1 mM IPTG), while the same strain with an empty plasmid showed an MIC of 4 μg/mL at both IPTG concentrations. SHuffle showed the same general trend but “skipped wells” when carrying an empty plasmid as further discussed below. However, in the presence of IPTG, MIC values for strains with the empty plasmid are generally 2-fold lower compared to strains without the plasmid (Table 2).

**TABLE 2:** MICs of E. coli B-strains carrying pET17b (“empty plasmid”) and pET17b-*mcr-3*-*FLAG* (“mcr-3”) after induction with different IPTG concentrations

Strain	Plasmid	MIC (μg/mL) obtained with^*a*^		
		0 mM IPTG	0.4 mM IPTG	1 mM IPTG
BL21 (DE3)	No plasmid	8	-	-
	empty plasmid	4	4	4
	mcr-3	4	8	8
T7 express lysY/l^q^	No plasmid	16	-	-
	empty plasmid	16	8	8
	mcr-3	16	16	16
SHuffle T7 express	No plasmid	16	-	-
	empty plasmid	16	0.5/8^*b*^	0.5/16^*c*^
	mcr-3	16	16	16

a MIC values represent the majority of three biological replicates; - indicates samples for which no MIC data were collected.

b One replicate each skipped one (0.5 μg/mL) or two wells (0.5 and 1 μg/mL).

c All three replicates skipped two wells (0.5 and 1 μg/mL).

We confirmed MCR-3-FLAG protein expression by Western blot analysis after the induction of expression in B-strains BL21(DE3), T7, and SHuffle with 0.4 mM IPTG as well as in the uninduced control (Fig. S2); semiquantitative analysis of MCR-3-FLAG levels shows that relative MCR-3-FLAG levels in strains induced with IPTG only showed a small increase compared to strains grown without IPTG (Fig. S2). These data suggest that leaky expression of *mcr-3-FLAG* occurs and is consistent with the observation that for two of the three B-strains (i.e., T7 and SHuffle), the MIC for colistin of the strains carrying pET17b-*mcr-3* was the same regardless of IPTG induction (Table 2).

### E. coli SHuffle carrying pET17b empty plasmid skips wells in MIC assay.

As part of the colistin MIC determination experiments detailed in the preceding section, we observed that B-strain SHuffle carrying the empty pET17b plasmid skips wells in MIC assays when induced with 0.4 or 1 mM IPTG (Table 2). The skipping wells phenotype, was more prominent with 1 mM IPTG, as all three biological replicates skipped two wells (i.e., 0.5 and 1 μg/mL) but continued to grow at 2, 4, and 8 μg/mL (Table 2), while for 0.4 mM, our replicates skipped one (i.e., 0.5 μg/mL) or two wells (i.e., 0.5 and 1 μg/mL), but all grew at 2 and 4 μg/mL. This observation is important as misinterpretation of skipped wells may provide inaccurate MIC values that could ultimately be misinterpreted as colistin susceptibility.

### E. coli B-strains encoding T7 RNA polymerase and transformed with pET17b show reduced cell viability in the presence of IPTG.

**FIG 2:**
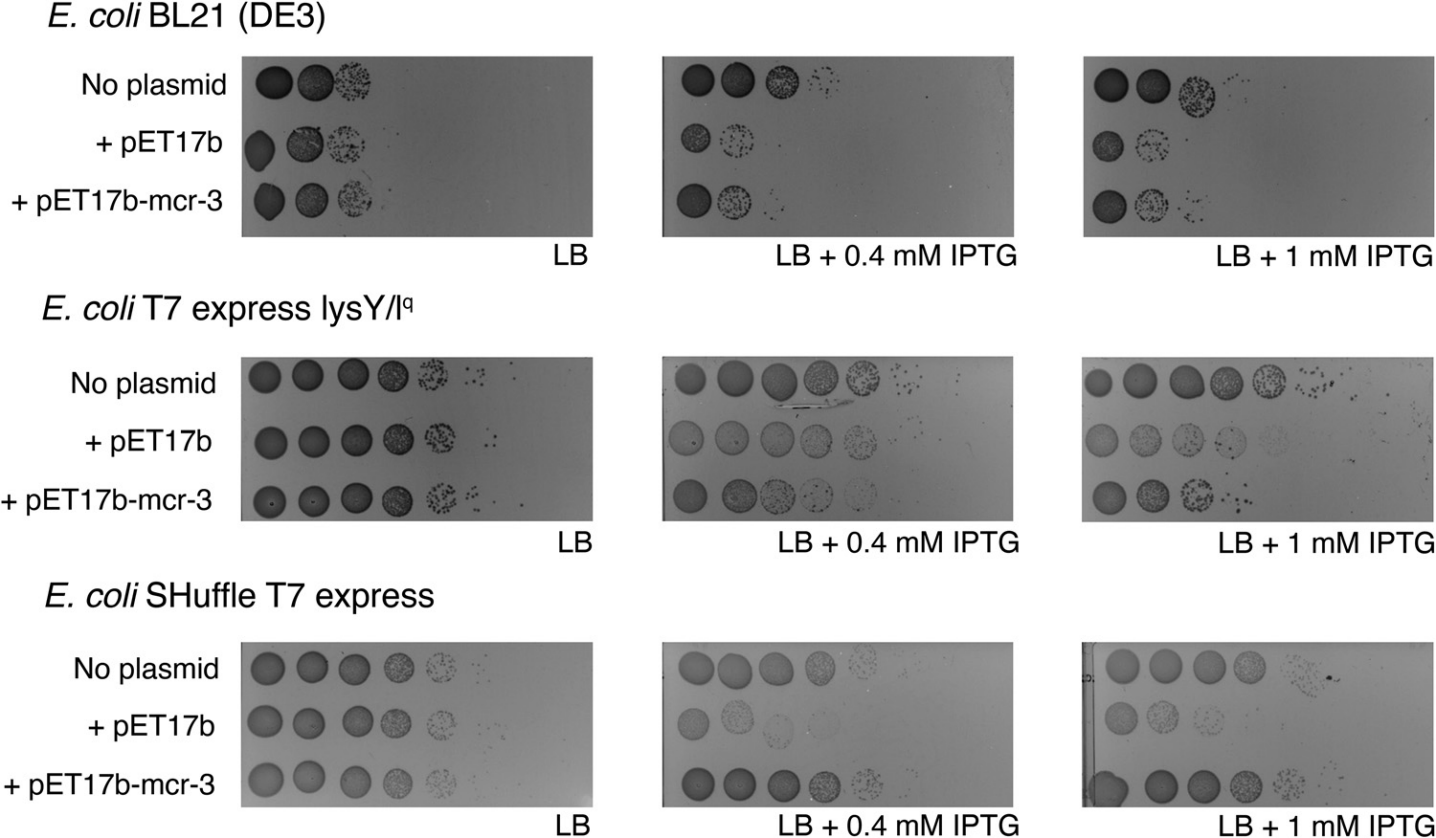
Cell viability of E. coli B-strains encoding T7 RNA polymerase on LB agar containing IPTG. Plating assay of E. coli B-strains without plasmid (“no plasmid”), with empty pET17b (“+ pET17b”), and with pET17b-*mcr-3* (“+ pET17b-mcr-3”) on 0, 0.4, or 1 mM IPTG (0 mM IPTG plates were prepared by adding distilled water instead of IPTG). Exponentially grown strains were 10-fold serial diluted in PBS and 10 μL volumes of undiluted cultures to 10^−7^ dilutions were spot plated. Results were consistent across three replicates and one replicate is shown here; images were acquired with the Bio-Rad ChemiDoc MP Imaging system.

In addition to skipped wells, cytotoxicity of either plasmid constructs and/or specific cloned genes (e.g., *mcr*) can yield misleading data that can provide inaccurate MIC values and hence lead to incorrect conclusions on resistance phenotypes conferred by a given overexpressed gene (40, 41). As previous reports indicated that overexpression of *mcr* genes may be toxic to the E. coli host cells (40, 41), we performed experiments to determine whether T7 RNA polymerase encoding E. coli B-strains transformed with pET17b-*mcr-3* show reduced cell viability. For these experiments, B-strains without plasmid (“no plasmid”), with pET17b, and with pET17b-*mcr-3* were plated on LB agar supplemented with 0, 0.4 and 1 mM IPTG (Fig. 2). On LB that was not supplemented with IPTG, growth patterns were indistinguishable for each given B-strain, no matter if they carried no plasmid, pET17b, or pET17b-*mcr-3* (Fig. 2). On the other hand, on LB supplemented with 0.4 or 1 mM IPTG, both BL21(DE3) and T7 showed decreased cell numbers when carrying either pET17b or pET17b-*mcr-3*, compared to the control without IPTG (Fig. 2). Interestingly, for E. coli SHuffle, the strain carrying pET17b also showed reduced growth, compared to the control without IPTG, while the strain carrying pET17b-*mcr-3* did not show reduced cell viability relative to the control without IPTG. In addition, T7 and SHuffle strains carrying pET17b show a different colony morphology on LB with 0.4 or 1 mM IPTG, compared to the LB control without IPTG; in the presence of IPTG these strains predominantly show tiny colonies (compared to colony sizes observed in the absence of IPTG) with only a few colonies showing similar sizes to the colonies observed when these strains were grown in the absence of IPTG (Fig. 2). Colony size is inversely proportional to toxicity, making it an additional indicator of toxicity (42). As reduced cell viability is observed in strains that are exposed to IPTG and carry an empty plasmid, this phenotype clearly does not solely reflect toxicity associated with *mcr* overexpression.

**FIG 3:**
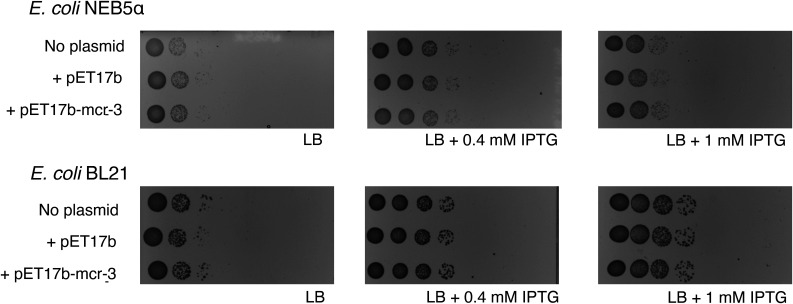
Cell viability of E. coli strains without T7 RNA polymerase on LB agar containing IPTG. Plating assay of E. coli strains without T7 RNA polymerase without plasmid (“no plasmid”), with empty pET17b (“+ pET17b”), and with pET17b-*mcr-3* (“+ pET17b-mcr-3”) on 0, 0.4, or 1 mM IPTG (0 mM IPTG plates were prepared by adding distilled water instead of IPTG). Exponentially grown strains were 10-fold serially diluted in PBS and 10 μL volumes of undiluted cultures to 10^−7^ dilutions were spot plated. Results were consistent across three replicates and one replicate is shown here; images were acquired with the Bio-Rad ChemiDoc MP Imaging system.

To determine whether the growth defect of B-strains that carry pET17b—either empty or expressing *mcr-3*—supplemented with IPTG is due to plasmid toxicity or due to combined effect of the plasmid and T7 RNA polymerase expression, we transformed pET17b and pET17b-*mcr-3* into BL21 and NEB5α, a B- and K-12 strain, respectively. These two strains do not carry a copy of T7 RNA polymerase and hence are not capable of using the T7 promoter that controls expression of recombinant genes in pET17b. When we plated BL21 and NEB5α (each without plasmid, with pET17b or pET17b-*mcr-3*) on LB agar with 0, 0.4, and 1 mM IPTG, bacterial numbers (as well as colony morphology) were indistinguishable in the presence and absence of IPTG regardless of whether a given strain carried no plasmid, pET17b, or pET17b-*mcr-3* (Fig. 3).

**FIG 4:**
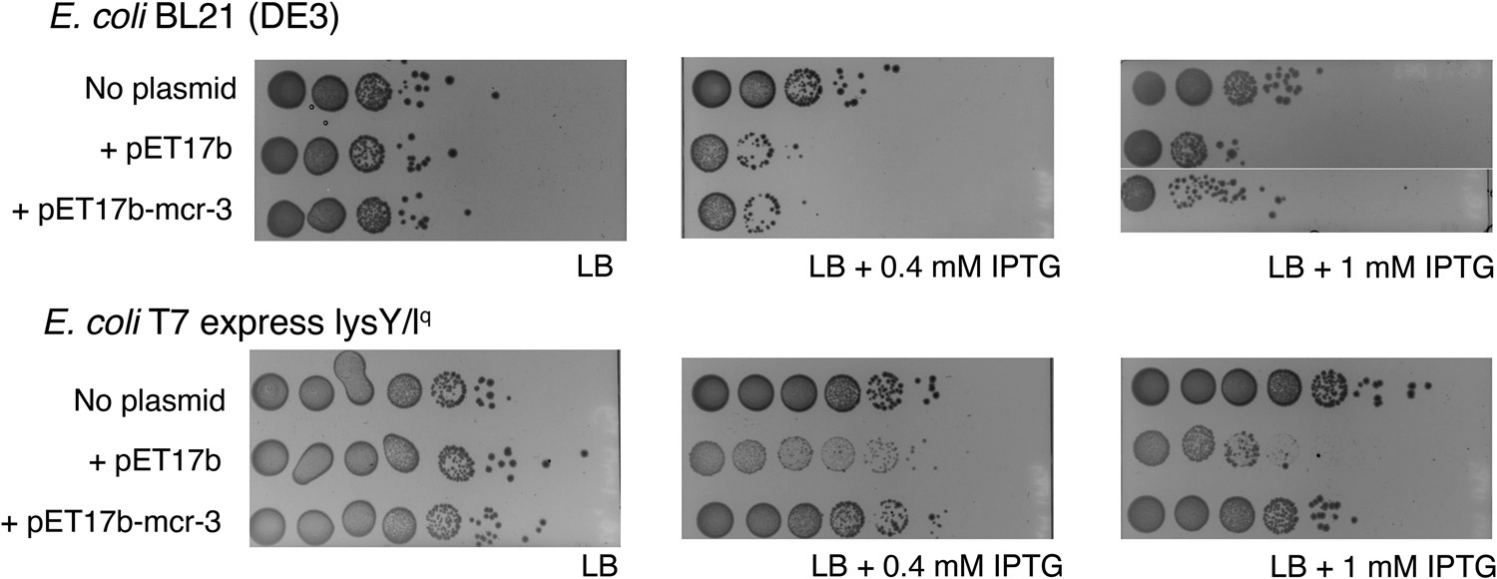
Cell viability of E. coli BL21(DE3) and T7 on LB agar containing 1 mM MgCl_2_ and IPTG. Plating assay of exponentially growing E. coli B strains BL21(DE3) and T7 without plasmid (“no plasmid”), with empty pET17b (“+ pET17b”), and with pET17b-*mcr-3* (“+ pET17b-mcr-3”) on LB containing 1 mM MgCl_2_ and 0, 0.4, or 1 mM IPTG (0 mM IPTG plates were prepared by adding distilled water instead of IPTG). Exponentially grown strains were 10-fold serially diluted in PBS and 10 μL volumes of undiluted cultures to 10^−7^ dilutions were spot plated. Results were consistent across three replicates and one replicate is shown here; images were acquired with the Bio-Rad ChemiDoc MP Imaging system.

As the toxicity seems limited to strains carrying T7 RNA polymerase, we hypothesized that T7 RNA polymerase expression may cause metabolic burden or interfere with specific cellular processes or cellular homeostasis. It has been previously shown that overexpression of T7 RNA polymerase may reduce magnesium availability, as it uses magnesium as a cofactor (43). We thus used LB agar supplemented with 1 mM MgCl_2_ to repeat cell viability experiments with two B-strains encoding T7 RNA polymerase (i.e., BL21[DE3]; T7) with either no plasmid, pET17b, or pET17b-*mcr-3* in the presence of 0, 0.4, or 1 mM IPTG. Our results show that MgCl_2_ does not appear to alleviate the cell viability effects seen when adding IPTG to pET17b carrying B-strains (Fig. 4), as we see reduced bacterial numbers for both BL21(DE3) and T7 (carrying pET17b or pET17b-*mcr-3*), similar to the patterns observed in LB not supplemented with MgCl_2_ (Fig. 4). Similarly, T7 carrying pET17b also showed the same atypical colony morphology (i.e., predominantly tiny colonies) on LB supplemented with MgCl_2_ as was observed on LB not supplemented with MgCl_2_. Our results suggest that the decrease in cell numbers is not associated with reduced availability of cellular magnesium, but rather suggest that cytotoxicity may be associated with the metabolic burden caused by the overexpression of T7 RNA polymerase.

## DISCUSSION

While antibiotic resistance determination (e.g., MIC determination) on clinical and other wild-type strains remains a cornerstone of antimicrobial resistance surveillance, heterologous expression of AMR genes represents another important approach for AMR resistance characterization. Advantages and applications of heterologous expression of AMR genes include (i) ability to characterize resistance conferred independent of genetic background (which may affect resistance gene expression, e.g., if AMR genes expression is tightly regulated and does not occur in wild-type strains grown under lab testing conditions), and (ii) ability to characterize resistance phenotypes if wild-type strains are not available. With widespread global use of Culture-independent diagnostic tests (CIDT) and WGS technologies (and a corresponding decrease of routine wild-type strain MIC determinations), use of novel approaches to identify and characterize emerging AMR genes (e.g., synthesis of genes found in genome databases, followed by characterization in heterologous expression hosts) is becoming more important. Importantly, similar approaches are already being used, including for the characterization of *mcr* genes (22, 24, 28, 40, 44).

A lack of standardization of these methods, including the use of different heterologous expression hosts, however, represents a major challenge. Here, we show that (i) commonly used heterologous expression hosts (i.e., E. coli B-strains) show intrinsic resistance to colistin, and (ii) commonly used overexpression systems (i.e., IPTG induction of pET17b plasmids in B-strains encoding T7 RNA polymerase) can induce cytotoxicity and other aberrant phenotypes (i.e., skipped wells), which is expected to lead to erroneous MIC results. While our findings indicate that E. coli B-strains should not be used as hosts for *mcr* variant MIC determinations, we also showed that E. coli K-12 strains appear to be appropriate hosts for characterization and identification of *mcr* genes. More broadly, our findings suggest that improved evaluation and standardization of heterologous expression system-based AMR determinations is urgently needed, particularly as use of CIDT based diagnostics increases.

### Commonly used heterologous expression hosts (i.e., E. coli B-strains) show intrinsic resistance to colistin, likely aided by mutations in *pmrAB*.

We found that four *E. coli* B-strains, BL21, BL21(DE3), T7, and SHuffle are intrinsically resistant to colistin with MICs of 8 to 16 μg/mL. This observation was surprising, as previous studies have used B-strains like BL21(DE3) and SHuffle to report MIC values for *mcr* variants and indicated that the strains were susceptible to colistin (25, 29, 30). However, our findings are consistent with studies by Trent et al. (37) and Xu et al. (36), who reported that B-strains BLR(DE3) (obtained from Novagen) and BL21(DE3) (obtained from both Novagen and Stratagene), respectively, were resistant to colistin. Trent et al. (37) attributed the colistin resistance of BLR(DE3) to phosphoethanolamine and l-Ara4N modifications of the lipid A portion of LPS as supported by the observation, based on analyzing ^32^P-labeled lipid A species, that colistin resistant Salmonella enterica Typhimurium *pmrA* mutant and E. coli BLR(DE3) showed similar lipid A modifications, which were different from colistin susceptible E. coli K-12 strain NovaBlue(DE3) (37). Overall, our data combined with these previous findings suggest that B-strains are not suited as heterologous expression hosts for characterization and identification of *mcr* genes. While prior evidence of colistin resistance in E. coli B-strains has been reported by Trent et al. (37) in 2001, this previous paper had focused on characterization of l-Ara4N transferase (ArnT) and thus appears to have been largely ignored as a substantial number of subsequent studies (25, 29, 30) still used E. coli B-strains for heterologous expression of *mcr* in order to identify and characterize *mcr* genes.

Comparative genomic analysis of the four E. coli B-strains used here, as well as two K-12 strains, NEB5α and MG1655, identified one nonsynonymous change in each *pmrA* and *pmrB*. Specifically, B-strains encode a glycine at amino acid site 29 in PmrA and a lysine at amino acid site 121 in PmrB, while K-12 strains encode a serine and a glutamate, respectively, at these locations. Mutations in the two-component system PmrAB represent a plausible mechanism for colistin resistance, as PmrAB regulates the expression of lipid A modification enzymes, and many studies have described mutations in *pmrAB* that impact colistin resistance (7, 12–15, 36, 45). The S29G change in PmrA has previously been identified in two colistin resistant E. coli isolates from chicken feces (13). These isolates also had one nonsynonymous mutation in *pmrB* and the authors did not experimentally confirm whether the S29G change in PmrA conferred resistance on its own (13). Future experimental work is needed to determine whether this change affects the expression of the lipid A modification system and, thus, colistin resistance. On the other hand, the E121K change in PmrB has been identified in multiple studies with different conclusions as to whether this change confers colistin resistance or not (15, 36, 45). While one study (15) identified the E121K change in more than 10 isolates, they did not experimentally test whether this PmrB mutation conferred resistance. Another study (45) classified this mutation as deleterious, using the PROVEAN tool, which predicts the effect of amino acid substitutions on protein function (46). Xu et al. identified the same change in PmrB in laboratory E. coli BL21(DE3) strains acquired from Stratagene and Novagene (36) and showed that colistin resistance increased when *pmrB* with at least 126 bp of the 3′ untranslated region (UTR) was expressed in an E. coli Top10 strain. However, cloning of *pmrB* without the 3′ UTR or with a shorter 3′ UTR did not appear to confer colistin resistance, suggesting a possible contributing role of mRNA stability. Expression of *pmrB* with the 126 bp 3’UTR also resulted in phosphoethanolamine and l-Ara4N modifications of lipid A (36), which could be linked to the observed colistin resistance. Overall, previous data support that mutations in both PmrA and PmrB can confer colistin resistance. While it thus is plausible that the combination of amino acid changes in PmrA and PmrB are the reason why B-strains are resistant to colistin, additional experimental work would be necessary to prove causality. We however do not see these types of mechanistic studies as necessary for this paper, as our data, along with previous studies, sufficiently demonstrate that B-strains are resistant to colistin and should therefore not be used to identify and characterize *mcr* genes.

### Commonly used overexpression systems can induce cytotoxicity and other aberrant phenotypes, which is expected to lead to erroneous colistin MIC results.

Our study found evidence that induction of T7 RNA polymerase expression induces toxicity in strains that carry the expression plasmid pET17b without an insert. This was supported by the finding that B-strains that encode T7 RNA polymerase and carry the empty plasmid showed reduced growth in the presence of IPTG, compared to growth in media without IPTG, while strains that do not encode T7 RNA polymerase (i.e., BL21, NEB5α) did not show any differences in cell growth between media with and without IPTG. Our findings are consistent with a study (47) that reported a growth defect in E. coli BL21(DE3) carrying an empty plasmid, when induced with IPTG. The authors attributed the growth defect in those strains to the increased metabolic burden due to the expression of the selective marker and the maintenance of the plasmid (47). Importantly, in BL21(DE3), T7, and SHuffle, expression of T7 RNA polymerase is controlled through the addition of IPTG (https://www.neb.com/products/competent-cells/e-coli-expression-strains/e-coli-expression-strains) and induction of T7 RNA polymerase expression in strains carrying the empty pET17b plasmid has been reported to initiate transcription from the T7 promoter upstream of the multiple cloning site (MCS) even if no gene is cloned into the MCS (https://research.fredhutch.org/content/dam/stripe/hahn/methods/biochem/pet.pdf). While this transcription initiation would be expected to create a short transcript (and not a translated protein), it has been shown that the native T7 terminator in pET17b is not 100% efficient, which can lead to read-through transcription (48, 49). If read-through transcription occurs, the downstream ampicillin resistance gene, which encodes a β-lactamase, will be transcribed, translated, and secreted (32, 50). Previous studies have shown that the overexpression of secreted and membrane proteins can result in toxicity due to the formation of cytoplasmic aggregates because the pathways for protein production and secretion are saturated (51, 52). The increased burden of gene expression and saturation of the secretion machineries through read-through transcription could thus explain the toxicity we and others have seen in IPTG-induced B-strains encoding T7 RNA polymerase (e.g., BL21[DE3]) carrying the empty pET17b plasmid. To confirm whether our hypothesis is correct, a future experiment should assess the cell viability of empty pET carrying B-strains in the presence of IPTG when T7 RNA polymerase is deleted or catalytically inactivated.

Our data also support that the observed cytotoxicity in T7 RNA polymerase-expressing strains can provide aberrant colistin MIC patterns (even in empty pET17b carrying strains), which could be misinterpreted as the strain being susceptible to colistin. When BL21(DE3) or T7 strains that express *mcr-3* are compared to the same strains carrying pET17b, *mcr-3* seems to provide a 2-fold increase in resistance. However, if *mcr-3* expressing strains are compared to parent strains without pET17b *mcr-3* does not appear to increase resistance. This observation demonstrates why it is important to test the MICs of parent strains. Importantly, we also found evidence for toxicity in E. coli BL21(DE3) and T7 that carry pET17b-*mcr-3* when T7 RNA polymerase expression is induced by IPTG. This observation is consistent with previous studies showing that overexpression of heterologous *mcr* genes in lab strains causes cytotoxicity, presumably because MCR and/or MCR induced lipid A modifications disrupt the bacterial cell membranes and impact membrane stability (40, 41). As our Western blots showed that MCR-3-FLAG is expressed even without IPTG induction, most likely due to the leakiness of the *lac* promoter that controls expression of T7 RNA polymerase (53, 54), our results suggest that *mcr-3* expression is only toxic in BL21(DE3) and T7 when expression occurs at higher levels. Overall, these data show that a number of potential direct and indirect factors affecting cytotoxicity phenotypes can occur when using T7 RNA polymerase encoding parent strains and pET overexpression plasmids to phenotypically assess different *mcr* genes.

In addition to the cytotoxicity phenotypes detailed above, we also found that SHuffle strains carrying the empty plasmid skip wells in MIC assays, which was not observed with BL21(DE3) and T7. This phenomenon of “skipped wells” is known to occur in Enterobacter spp. in response to colistin (55, 56) and has also been reported recently by Kananizadeh et al. (57) when testing wild-type isolates carrying *mcr-9* in MHII broth supplemented with peptone, tryptone or casein. To our knowledge we are the first to report the phenomenon when testing *mcr* genes or their empty expression plasmids in heterologous expression strains grown in standard MHII broth. “Skipping wells” in broth microdilution is a sign that strains may be heteroresistant; however, further assessment through population analysis profiling (PAP) is needed to confirm heteroresistance (58). According to CLSI guidelines, MICs of strains that skip two or more wells in BMD assays should not be reported (59), however, it is plausible that skipped wells may be misreported as a lower MIC than the “true MIC.”

Based on the cytotoxicity phenotypes observed with both IPTG induction of B-strains encoding T7 RNA polymerase carrying the empty pET17b plasmid and with overexpression of *mcr-3* as well the “skipped well” phenomenon observed for SHuffle strains carrying pET17b, we suggest that the commonly used expression system of B-strains with T7 RNA polymerase and pET plasmids may not be appropriate to phenotypically characterize *mcr* genes (and possibly any antimicrobial resistance genes). Specifically, these aberrant phenotypes could lead to misinterpretation of colistin MIC values, particularly when using CLSI cutoff values to determine resistance (35). However, our experiments cannot explain why other studies have reported that the parental B-strains that carry T7 RNA polymerase but do not carry plasmids are susceptible to colistin (29, 30); for example, a study (29) has reported a colistin MIC value of 0.5 μg/mL for E. coli SHuffle T7 express, while we found a MIC value of 16 μg/mL. Among other reasons, this could be caused by colistin preparations with reduced efficacy, lower cell density than used here and recommended by CLSI (59), pregrowth of strain under nonstandard conditions, and possibly even use of parent strains that did not have the colistin resistance phenotype reported here (e.g., due to mutations, contamination with other E. coli strains, or sharing of mislabeled or misassigned strains between labs).

### While E. coli B-strains should not be used as hosts for colistin MIC determinations, E. coli K-12 strains appear to be appropriate hosts for characterization and identification of *mcr* genes.

We found that two E. coli K-12 strains, NEB5α and Top10, were susceptible to colistin with MICs of <0.5 μg/mL. Our findings of K-12 strain’s MIC values are consistent with a previous study that reported that K-12 strain NovaBlue(DE3) is susceptible to colistin and has no lipid A modifications (37). Other studies have used E. coli DH5α (18, 20, 26, 60)—the NEB5α strain that was used here is a derivative of DH5α according to NEB (https://www.neb.com/products/c2987-neb-5-alpha-competent-e-coli-high-efficiency#Product%20Information)—and E. coli Top10 (27, 61, 62) as heterologous expression strains for *mcr* variants and reported similar colistin MICs of their heterologous expression strains. The observed colistin sensitivity patterns are consistent with our genotypic findings of only one synonymous base change in *pmrB* and no change in *pmrA* of NEB5α in comparison to E. coli MG1655. Based on our phenotypic and genotypic observations, E. coli K-12 strains appear to be appropriate heterologous expression strains for the characterization and identification of *mcr* variants. Our findings also emphasize that it is essential to perform an initial MIC screen with strains and plasmids that are being considered heterologous expression hosts, regardless of which antibiotic resistance gene is being characterized. Based on previous studies, expression plasmids pCR (27), pBADb (61), pUC19 (18), and native *mcr* carrying plasmids (26) have been used in conjunction with E. coli K-12 strains, DH5α and Top10, to identify novel *mcr* variants and could provide alternatives to pET plasmids. As E. coli K-12 strains DH5α and Top10 do not encode T7 RNA polymerase these strains cannot be used in combination with pET plasmids. Overall, our findings suggest that improved evaluation and standardization of heterologous expression system-based AMR determinations are urgently needed.

## MATERIALS AND METHODS

### Strains, plasmids, and growth conditions.

The strains and plasmids used in this study are shown in Table 3. All S. enterica strains and E. coli strains were grown at 37°C with shaking at 200 rpm, in Difco LB Lennox Broth (LB; Becton, Dickinson and Company [BD]; Franklin Lakes, NJ; cat. number 240230). For E. coli strains carrying the pET17b plasmids, 100 μg/mL of ampicillin (AMP) was added to maintain the plasmid. All plasmids were transformed into competent E. coli strains by heat shock, according to the manufacturer’s recommendations (New England Biolabs [NEB]; Ipswich, MA).

**TABLE 3:** Strains and plasmids used in the study

ID name	Description	Manufacturer (cat. number)	Abbreviation
Strain [lineage]			
E. coli NEB5α [K-12]	fhuA2Δ(argF-lacZ)U169 phoA glnV44 Φ80Δ(lacZ)M15 gyrA96 recA1 relA1 endA1 thi-1 hsdR17	NEB (C2987)	NEB5α
E. coli Top10 [K-12]	F- mcrA Δ( mrr-hsdRMS-mcrBC) Φ80lacZΔM15 Δ lacX74 recA1 araD139 Δ( araleu)7697 galU galK rpsL (StrR) endA1 nupG	ThermoFisher (C404010)	Top10
E. coli BL21 [B]	fhuA2 [lon] ompT gal [dcm] ΔhsdS	NEB (C2530)	BL21
E. coli BL21 (DE3) [B]	fhuA2 [lon] ompT gal (λ DE3) [dcm] ΔhsdS λ DE3 = λ sBamHIo ΔEcoRI-B int::(lacI::PlacUV5::T7 gene1) i21 Δnin5	NEB (C2527)	BL21(DE3)
E. coli T7 express lysy/Iq [B]	MiniF lysY lacI^q^(Cam^R^) / fhuA2 lacZ::T7 gene1 [lon] ompT gal sulA11 R(mcr-73::miniTn10–Tet^S^)2 [dcm] R(zgb-210::Tn10–Tet^S^) endA1 Δ(mcrC-mrr) 114::IS10	NEB (C3013)	T7
E. coli T7 SHuffle express [B]	fhuA2 lacZ::T7 gene1 [lon] ompT ahpC gal λatt::pNEB3-r1-cDsbC (Spec^R^, lacI^q^) ΔtrxB sulA11 R(mcr-73::miniTn10–Tet^S^)2 [dcm] R(zgb-210::Tn10 –Tet^S^) endA1 Δgor Δ(mcrC-mrr)114::IS10	NEB (C3029)	SHuffle
S. enterica FSL R9-3269	S. enterica Typhimurium containing *mcr-3*	n/a^*a*^	
S. enterica FSL R9-5409	S. enterica Typhimurium, colistin susceptible	n/a^*a*^	
Plasmid			
pET17b	pET17b plasmid (IPTG/lactose inducible promoter, Amp resistance)	Novagen (69663)	pET17b
pET17b-*mcr-3-FLAG*	*mcr-3* gene + 3× FLAG tag fusion	n/a^*b*^	pET17b-*mcr-3*

a Not applicable, strains were obtained from our lab’s internal strain collection. Information about the strains can be found on foodmicrobetracker.net.

b Not applicable, plasmid was constructed in this paper.

### Cloning of *mcr-3.1*.

*mcr-3.1* was amplified via PCR with the primers pET17b_mcr3_F and pET17b_mcr3_R (Table 4) from the genomic DNA of isolate S. enterica FSL R9-3269. The PCR amplicon was purified and used as a template to add the 3× FLAG tag through primer walking using the primers pET17b_mcr3_F and pET17b_3×_FLAG_R2 (Table 4). All PCRs were performed using Q5 high-fidelity DNA polymerase (NEB, cat. #M0491S) according to the manufacturer’s recommendations. PCR products and the pET17b plasmid (Novagen [EMD Millipore], cat. #69663) were digested with restriction enzymes NdeI (NEB, cat. number R0111S) and BamHI (NEB, cat. number R0136S), purified, and ligated with T4 DNA ligase (NEB, cat. number M0202L) according to the manufacturer’s recommendations. Ligation products were transformed via heat shock into E. coli Lemo21(DE3) (NEB, cat. number C2528) according to the manufacturer’s recommendations. Transformants were plated on LB + 100 μg/mL ampicillin (Amp) agar plates and incubated overnight growth at 37°C. Successful constructs were confirmed via Sanger sequencing and subsequent analysis in Geneious Prime (Auckland, New Zealand). Plasmid DNA from confirmed clones was purified using the GeneJet Plasmid Miniprep kit (ThermoFisher Scientific; Waltham, MA; cat. number K0503) and transferred into E. coli SHuffle T7 express (NEB, cat. number C3029), T7 express lysY/lq (NEB, cat. number C3013), BL21(DE3) (NEB, cat. number C2527), BL21 (NEB, cat. number C2530), and NEB5α (NEB, cat. number C2987) via heat shock as described above.

**TABLE 4:** Primers used in this study

Primer name	DNA sequence (5′->3′)	Purpose
pET17b_mcr3_F	CGCGCATATGAGTGATGTCTCGTTAGAAAGTGATTGTTGGA	*mcr-3* amplification from R9-3269
pET17b_mcr3_R	CATCATGATCTTTATAATCTGGACGTTGAACATTACGACATT GACTGAAAATATCT	*mcr-3* amplification from R9-3269
pET17b_3×-FLAG-R2	CGCGGATCCTTATTTATCATCATCATCTTTATAATCAATATCA TGATCTTTATAATCACCATCATGATCTTTATAATCTGGACG	Addition of 3 × FLAG tag to *mcr-3*
AG67-22-pet16B-F	CGCGAAATTAATACGACTCACTA	pET17b sequencing primer forward
AG76-113	GCCCTCGAGGGCCCTTTCGTCTTCAAGAAT	pET17b sequencing primer reverse

### Colistin susceptibility testing.

Colistin susceptibility was tested by broth microdilution (BMD) according to the Clinical and Laboratory Standards Institute (CLSI) recommendations (59), except that PlateOne polypropylene 96-well plates (USAScientific; Ocala, FL; cat. #1837-9610) were used instead of polystyrene plates due to the known higher binding affinity of colistin to polystyrene (63, 64). Briefly, strains were grown in LB (+ 100 μg/mL Amp for pET17b plasmid carrying strains) at 37°C and 200 rpm shaking for 12 to 18 h and subsequently diluted 1:200 in BBL Mueller-Hinton II broth cation-adjusted (MHII, BD cat. number 212322) (+ 100 μg/mL Amp for pET17b plasmid carrying strains), followed by incubation at 37°C and 200 rpm shaking until cultures reached an OD_600_ of 0.3 to 0.4. Strains carrying pET17b plasmids were induced by the addition of 0.4 mM IPTG, 1 mM IPTG or filter-sterilized dH2O as a negative-control and grown for another 2 h. E. coli BL21(DE3) and T7 express lysY/l^q^ were induced at 37°C and 200 rpm shaking, while E. coli SHuffle T7 express was induced at 30°C, 200 rpm, based on the manufacturer’s recommendations (NEB). Strains were inoculated at a concentration of 5 × 10^5^ CFU/mL in MHII broth containing colistin (0.5 to 128 μg/mL) and IPTG (0, 0.4, or 1 mM) and grown statically for 16 to 20 h at 35°C. S. enterica isolates R9-5409 and R9-3269 were used as controls, because R9-5409 does not encode any *mcr* variant and R9-3269 natively encodes *mcr-3.1*; their MIC values were previously reported as 0.5 μg/mL and 4 μg/mL, respectively (22).

### Alignment of *pmrA* and *pmrB* genes.

The DNA sequences of E. coli str. K-12 substr. MG1655 genes *pmrA/basR* (locus tag: b4113) and *pmrB/basS* (locus tag: b4112) were downloaded from NCBI (Accession No. NC_000913.3). The *pmrA* and *pmrB* genes encoded by strains used in this paper were identified using BLAST (65), by querying the sequences against the genomes of NEB strains E. coli BL21 (Accession No. CP053601), BL21(DE3) (Accession No. CP053602), T7 express lysY/l^q^ (Accession No. CP053595), SHuffle T7 Express (Accession No. CP014269), and NEB5α (Accession No. CP017100.1). The identified *pmrA* and *pmrB* sequences were downloaded and converted into amino acid sequences using ExPASy, and both DNA and amino acid sequences were aligned using Clustal Omega (66, 67).

### Western blot analysis of MCR-3-FLAG.

To confirm MCR-3-FLAG expression, E. coli B-strains containing the pET17b and pET17b-*mcr-3-FLAG* plasmid were grown at 37°C and 200 rpm shaking in LB + 100 g/mL Amp for 12 to 18 h, followed by 1:200 backdilution in MHII broth + 100 g/mL Amp and incubation until an OD_600_ of 0.3 to 0.4 was reached. Cells were induced with 0.4 mM IPTG or dH_2_O for 2 h and collected by centrifugation at 7,197 × *g* for 10 min (Sorvall X4RF PRO-MD centrifuge). Cells were resuspended in SDS-PAGE lysis buffer and lysed by 2 rounds of sonication using Branson Sonifier 50 sonicator (80% duty cycle, 7 output control) for 30 s on ice. Lysed cells were resolved on a 4% to 20% Mini-Protean TGX precast protein SDS-PAGE gel (Bio-Rad Laboratories; Hercules, CA, cat. number 4568096). Resolved protein bands were visualized by activating the prestained SDS-PAGE gels with 1.5 min UV light exposure using the Bio-Rad ChemiDoc MP Imaging system. The proteins were transferred to polyvinylidene difluoride (PVDF) membrane using the Trans-Blot Turbo RTA Transfer kit, PVDF (Bio-Rad Laboratories, cat. number 170-4272) and the Trans-Blot Turbo transfer system (Bio-Rad Laboratories), according to the manufacturer’s recommendations. The membrane was blocked with TTBS (TBS buffer [50 mM Tris-Cl, 150 mM NaCl, pH 7.5] with 0.1% [vol/vol] Tween 20) containing 5% Blotting-Grade Blocker (Bio-Rad Laboratories, cat. number 170-6404) for 30 min, followed by incubation, with a rabbit anti-FLAG primary antibody (Sigma, cat. number F7425, at 1:500 dilution) diluted in TTBS with 0.5% (W/V) skimmed milk powder, at room temperature overnight with gentle shaking. The membrane was washed three times for 10 min with TTBS before incubating with goat anti-rabbit-horseradish peroxidase secondary antibody (Thermo Fisher Scientific, cat. number 65-6120, at 1:3,000 dilution) for 2 h at room temperature with gentle shaking. The membrane was washed three times for 10 min with TTBS and once for 10 min with TBS before developing it with the Clarity Western ECL Substrate (Bio-Rad Laboratories, cat. number 170-5061) and visualizing it using the Bio-Rad ChemiDoc MP Imaging system. Densitometric analysis of band intensity was carried out using the Bio-Rad Image Lab 6.1 software. Levels of MCR-3-FLAG expression were semiquantitively estimated by normalizing the area under the curve (AUC) of the MCR-3-FLAG band on the developed PVDF membrane to the sum of the AUCs for all protein bands in the corresponding lane on the UV-activated SDS-PAGE gel (“total amount of protein”).

### Plating assay.

Cell viability in response to IPTG addition was tested by monitoring cell growth on agar plates. Briefly, strains were grown in LB broth (strains containing pET17b plasmids and constructs were supplemented with 100 μg/mL Amp) for 12 to 18 h at 37°C and 200 rpm shaking. These cultures were used to inoculate MHII broth tubes at 1:200-fold dilution (strains containing pET17b were supplemented with 100 μg/mL). MHII broth culture tubes were incubated at 37°C and 200 rpm shaking until an OD_600_ of 0.3 to 0.4 was reached. Ten-fold serial dilutions were prepared from each culture in phosphate-buffered saline, and 10 μL volumes were spotted onto Difco LB Miller (Luria-Bertani) Broth (BD, cat. number 244620) agar plates (prepared with 15 g/L Bacto Agar [BD, cat. number 214010]), supplemented with 0 mM, 0.4 mM or 1 mM IPTG and/or 1 mM MgCl_2_. Plates were incubated at 37°C overnight. Pictures were taken with the Bio-Rad ChemiDoc MP imaging system. Cell viability was determined by comparing the highest dilutions with visible colonies and inspecting colony size and morphology.
