# Supplementary material for: Escherichia coli B-Strains Are Intrinsically Resistant to Colistin and Not Suitable for Characterization and Identification of mcr Genes
Source: Microbiol Spectr. 2023 May 18;11(3):e00894-23. doi: 10.1128/spectrum.00894-23 (PMC10269513; doi:10.1128/spectrum.00894-23)
Supplement: Supplemental file 1 — Fig. S1 and S2. Download spectrum.00894-23-s0001.pdf, PDF file, 0.7 MB [file spectrum.00894-23-s0001.pdf]

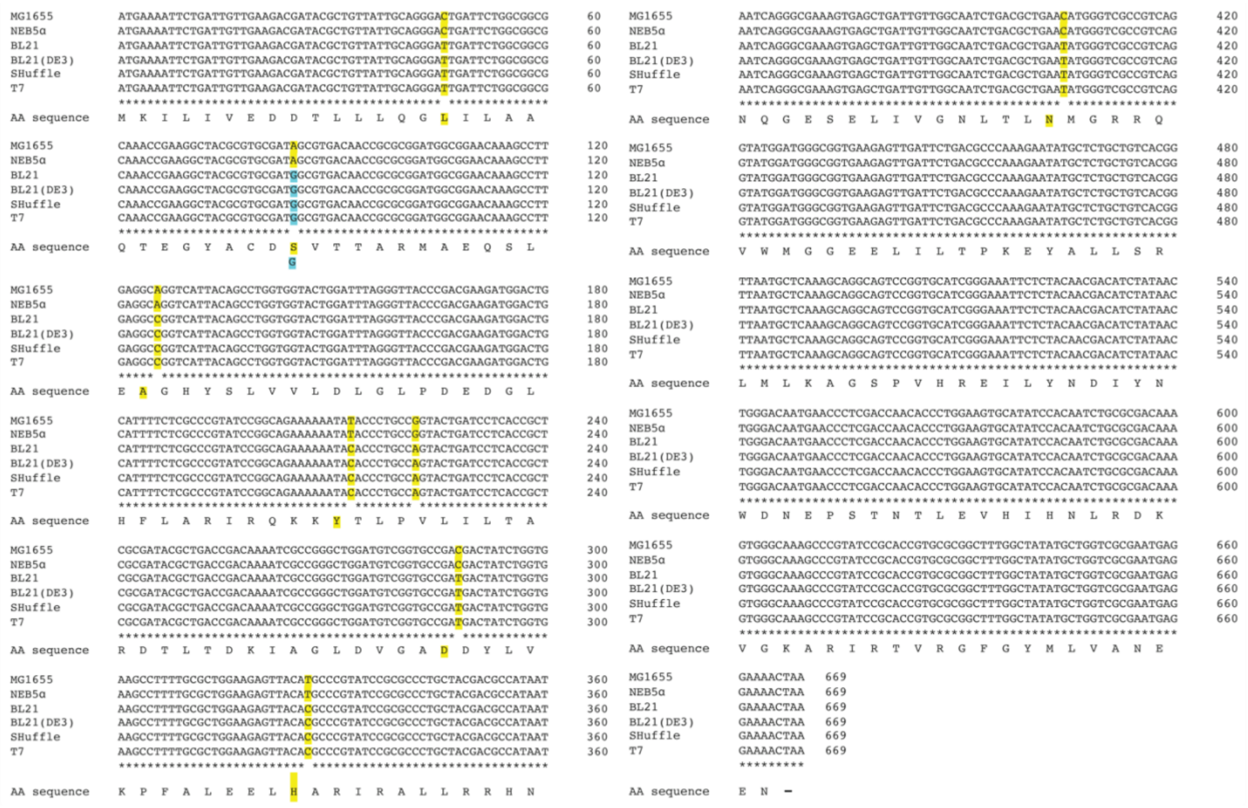

Supplemental Figure 1. Alignment of *pmrA* DNA sequences of *E. coli* B- and K-12 strains used in this study. Alignment of genes by Clustal Omega (66). Base changes are highlighted. The coloring indicates which base change correlates with which amino acid. For details on how sequences were acquired, refer to the materials & methods section. [T7 = T7 express lysY/I^{q}, SHuffle = SHuffle T7 express]

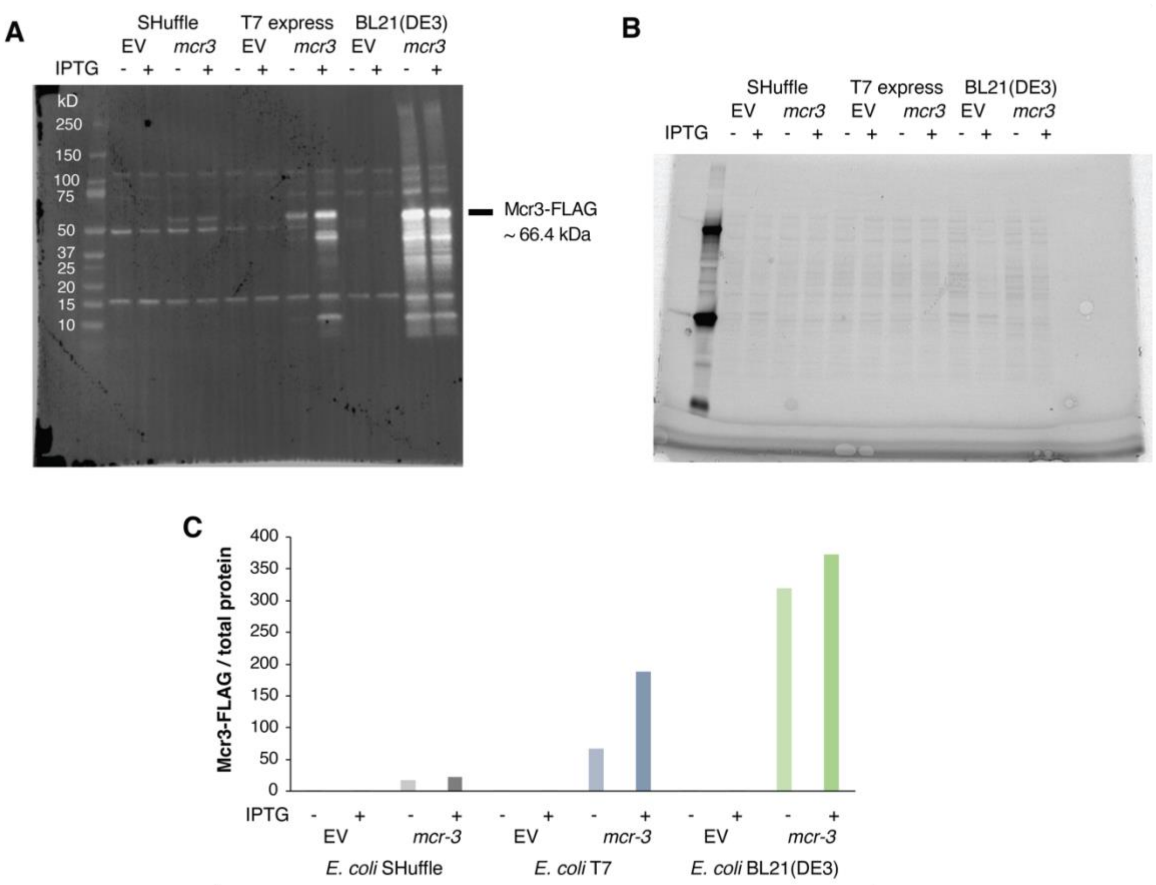

Supplemental Figure 2. **Anti-FLAG Western Blot for detection of MCR-3-FLAG in *E. coli* B-strains carrying T7 RNA polymerase. (A)** Detection of *mcr-3-FLAG* expression in *E. coli* B-strains with empty pET17b (“EV”) and pET17b-*mcr-3* (“mcr3”). Strains were isolated after 2 hours incubation with 0.4 mM IPTG (“+“) or dH2O (“-”). Rabbit anti-FLAG and goat anti-rabbit-horseradish peroxidase antibodies were used to tag proteins, followed by development with Bio-Rad Clarity Substrate. Pictures were obtained with the Bio-Rad ChemiDox MP Imaging system. **(B)** Total protein visualization before transfer to the membrane and antibody staining; images were acquired with the ”Stain Free Gel” setting of the Bio-Rad ChemiDoc MP Imaging system after activation of the SDS page gel with UV light for 1.5 mins. **(C)** Graph of *mcr-3-FLAG* expression levels in each of the strains based on densitometric analysis of total protein (B) and MCR-3-FLAG bands (A).

MCR-3-FLAG level shown on the Y axis were calculated by dividing the volume of the MCR-3-FLAG band from **A** through the volume of all bands in the corresponding lane from **B** obtained by densitometric analysis using the Bio-Rad Image Lab 6.1 software.

[SHuffle = SHuffle T7 express, T7 = T7 express lysY/I^q]
